# Potential Links Between Aging, Mitochondrial Dysfunction, and Drug Transporter Function—Molecular Mechanisms and Pharmacokinetic Implications

**DOI:** 10.3390/ijms27052206

**Published:** 2026-02-26

**Authors:** Patryk Rzeczycki, Oliwia Pęciak, Martyna Plust, Marek Droździk

**Affiliations:** Department of Experimental and Clinical Pharmacology, Pomeranian Medical University, 72 Powstańców Wielkopolskich Avenue, 70-111 Szczecin, Poland

**Keywords:** aging, pharmacokinetics, gastrointestinal tract, mitochondrial dysfunction, oxidative stress, P-glycoprotein (ABCB1), ABCG2, intestinal drug absorption, bioavailability, energy metabolism, elderly patients, polypharmacy, drug–drug interactions, geriatric pharmacotherapy

## Abstract

Aging is associated with complex physiological changes that influence drug pharmacokinetics, including alterations in mitochondrial function and gastrointestinal (GI) drug transporter activity. Mitochondrial dysfunction—characterized by reduced oxidative phosphorylation, mitochondrial DNA damage, and increased reactive oxygen species—is a hallmark of aging and may affect energy- and redox-dependent cellular processes in the gut. At the same time, aging can modulate the expression and function of key intestinal drug transporters from the ATP-binding cassette (ABC) and solute carrier (SLC) families, which play a central role in oral drug absorption and bioavailability. This review examines the molecular links between age-related mitochondrial dysfunction and regulation of GI drug transporters, with a focus on their pharmacokinetic consequences in older adults. We summarize evidence of mitochondrial decline in the aging intestine and discuss how mitochondrial signals—such as cellular energy status and oxidative stress—regulate transporter expression and activity via pathways including AMPK (AMP-Activated Protein Kinase), Sirtuin–FOXO (Forkhead box O transcription factors), Nrf2 (Nuclear factor erythroid 2-related factor 2), and NF-κB (Nuclear Factor kappa B). We highlight clinical examples of drugs showing age-related changes in bioavailability that may be attributable to transporter dysfunction. Finally, we discuss therapeutic implications for geriatric pharmacotherapy, including dose adjustment, management of transporter-mediated drug–drug interactions, and strategies aimed at preserving mitochondrial health.

## 1. Introduction

**Figure 1:**
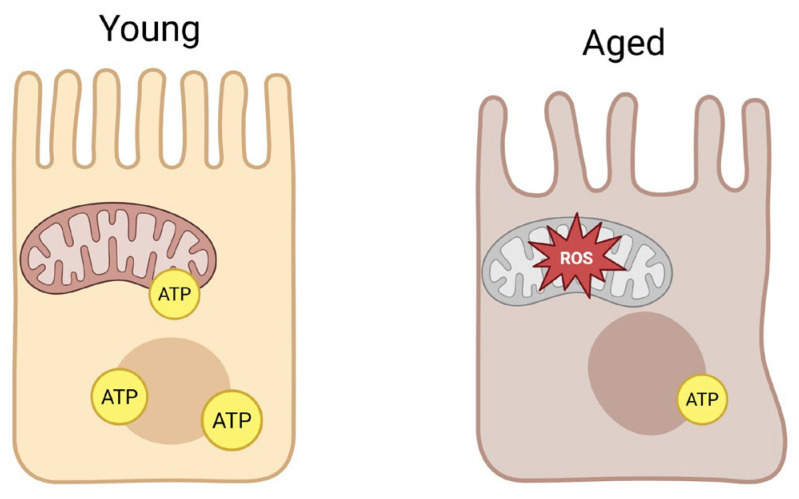
Young vs. aged intestine. The young gut is characterized by an intact epithelial barrier, with higher ATP production in enterocytes, lower ROS, and preserved tight junction dynamics resulting in efficient absorption and barrier function. On the contrary, the aged gut is characterized by impaired tight junctions, lower ATP production due to mitochondrial dysfunction, and higher ROS and oxidative stress, which leads to diminished barrier integrity and altered oral drug absorption among elderly patients. ATP—adenosine triphosphate.

The world’s population is aging, with a rapidly increasing proportion of individuals over 65 [1]. This demographic shift poses challenges for pharmacotherapy, as older patients often exhibit altered drug responses due to age-related physiological changes [2]. Key pharmacokinetic processes—absorption, distribution, metabolism, and excretion—can be affected by aging [3]. While declines in renal clearance and hepatic metabolic capacity are well-documented contributors to altered drug disposition in the elderly [4], the role of the gastrointestinal tract has garnered growing attention [5]. Aging can influence oral drug absorption through changes in GI anatomy and function, including reduced gastric motility and secretion, decreased intestinal surface area, and impaired epithelial integrity [6,7] (Figure 1). An often-underappreciated factor is the change in expression and activity of GI drug transporters with age [8]. These transporters—notably members of the ABC efflux pump family and SLC uptake carriers—control the influx and efflux of drugs across the intestinal epithelium, thereby governing bioavailability [9,10]. Even subtle age-related modulation of transporter function can alter plasma drug levels and clinical outcomes in older patients [11].

A parallel hallmark of aging is mitochondrial dysfunction [12]. Mitochondria play a pivotal role in cellular energy homeostasis, redox balance, and signaling; their decline with age has widespread consequences for organ function [13,14]. In tissues with high energy demand and turnover—such as the intestinal mucosa—age-related mitochondrial deficits are particularly impactful [15]. Research indicates that mitochondria in the aging gut exhibit decreased oxidative phosphorylation capacity, increased ROS production, and diminished ATP generation [16,17,18]. This mitochondrial insufficiency may impair energy-dependent cellular processes and activate stress response pathways [19]. Drug transporters, especially ABC pumps like P-glycoprotein (P-gp), require ATP to actively extrude substrates [20]. Thus, a decline in mucosal ATP supply or an increase in oxidative stress could directly affect transporter activity and regulation [21,22]. The convergence of mitochondrial aging and transporter function is an emerging area of research with significant pharmacological implications.

This review provides a comprehensive examination of the link between aging, mitochondrial dysfunction, and GI drug transporter function [23].

## 2. Mitochondrial Dysfunction in Aging

Mitochondrial dysfunction is a well-recognized feature of aging. In general, aging cells accumulate damage to mitochondrial DNA (mtDNA) and proteins, suffer impaired respiratory chain activity, and show altered dynamics (fission–fusion balance) and turnover (mitophagy) [24]. These changes result in a progressive decline in cellular energy production and redox homeostasis. The intestine is no exception: studies indicate that the aging gastrointestinal mucosa experiences significant mitochondrial functional deterioration. Models and human GI tissues demonstrate an age-associated reduction in electron transport chain efficiency and ATP output, accompanied by increased oxidative stress in enterocytes [25,26]. For instance, activities of key respiratory enzymes (such as cytochrome c oxidase and NADH dehydrogenase) were reported to be lower in older intestinal samples compared to young ones, suggesting a loss of mitochondrial respiratory capacity with age [27]. Meanwhile, levels of ROS and markers of oxidative damage (e.g., lipid peroxidation, protein carbonylation) tend to rise in the aging gut, reflecting an imbalance between ROS generation and antioxidant defenses. This oxidative milieu can trigger local inflammation and disrupt normal cellular signaling in the GI tract [28].

**Figure 2:**
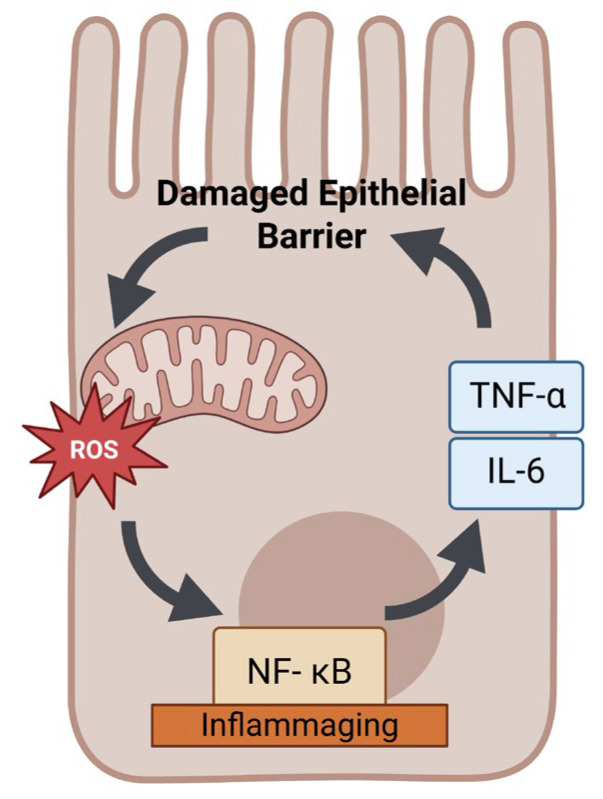
Inflammaging in the gut. Mitochondrial dysfunction elevates ROS, causing NF κB activation and promoting chronic low-grade inflammation (inflammaging). This inflammatory environment increases cytokines (TNF α, IL 6), impairs epithelial barrier integrity, and modulates drug transporter function, creating a vicious cycle that further exacerbates mitochondrial damage. ROS—reactive oxygen species; NF κB—nuclear factor kappa B; TNF α—tumor necrosis factor alpha; IL 6—interleukin 6.

One consequence of age-related mitochondrial changes in the gut is a decline in epithelial barrier function and regenerative capacity. Intestinal epithelial cells rely on mitochondrial ATP to fuel the ion pumps and tight junction dynamics that maintain barrier integrity [29]. In aging, mitochondrial ATP output is diminished, which may compromise these energy-intensive processes. Aged intestinal epithelia have slower turnover and an impaired stress response, partly due to mitochondrial deficits in intestinal stem cells and enterocytes [30]. Mitochondrial DNA mutations accumulating in colonic crypt stem cells can impair their function and contribute to the thinning and dysfunction of the colonic epithelium over time [31]. Additionally, excessive ROS from dysfunctional mitochondria can activate pro-inflammatory pathways (e.g., NF-κB) in the gut, promoting a chronic low-grade inflammation (“inflammaging”) environment. This local inflammation can further damage mitochondria, creating a vicious cycle [32] (Figure 2).

It is important to note that not all aspects of mitochondrial change are negative; some are compensatory. There is evidence of an adaptive upregulation of mitochondrial biogenesis signals (such as PGC-1α (Peroxisome Proliferator-Activated Receptor-γ Coactivator-1 alpha) and Nrf2) in certain contexts of aging, perhaps as the body’s attempt to counteract mitochondrial loss [33]. However, these compensatory mechanisms may be blunted in advanced age or overwhelmed by cumulative damage [34]. Lifestyle interventions like exercise and calorie restriction have been shown to mitigate age-related mitochondrial decline in the GI tract [35,36].

In summary, the aging GI tract is marked by mitochondrial dysfunction, characterized by reduced energy production and increased oxidative stress [37,38,39].

## 3. Gastrointestinal Drug Transporters (ABC and SLC Families)

The absorption of orally administered drugs is determined not only by passive diffusion across membranes but also by active and facilitated transport processes in the intestinal epithelium [40]. The human small intestine (and to a lesser extent the colon) expresses a variety of drug transporters that can either export compounds back into the lumen (efflux transporters) or import them into enterocytes (uptake transporters) [41]. Table 1 summarizes the principal transporters in the gut, their family classification, typical substrates, localization along the GI tract, and any known age-related changes in expression.

P-glycoprotein (P-gp) is expressed on the apical (luminal) surface of enterocytes throughout the intestine, with particularly high levels in the jejunum and ileum [42,43,44]. P-gp substrates are typically large, hydrophobic or amphipathic molecules, often cationic, and include many clinically important drugs (e.g., digoxin, anticancer agents like paclitaxel, calcineurin inhibitors, HIV protease inhibitors) [45]. By pumping such drugs back into the intestinal lumen, P-gp limits their net absorption and is a major factor in the oral bioavailability of P-gp substrates [46].

Other important ABC efflux transporters in the gut include Breast Cancer Resistance Protein (BCRP), encoded by ABCG2, and Multidrug Resistance-Associated Protein 2 (MRP2), encoded by ABCC2 [47,48,49]. BCRP is also located apically in enterocytes and has overlapping substrate specificity with P-gp [50].

MRP2 is an efflux pump that exports organic anions, including drug conjugates; it is apically expressed in the small intestine and colon [51]. Additionally, MRP3 (ABCC3) is present on the basolateral side of enterocytes, where it can efflux certain absorbed conjugates into blood, thus working in tandem with apical MRP2 [52].

SLC Uptake Transporters: Complementing the efflux pumps, the intestine also expresses uptake transporters that facilitate absorption of nutrients and drugs [53]. The most significant are the Organic Anion-Transporting Polypeptides (OATPs, gene family SLCO) and Peptide Transporter 1 (PepT1, SLC15A1) [54].

OATP2B1 (SLCO2B1) is considered the major OATP isoform in the human intestine. OATP2B1’s function can be influenced by dietary factors; for instance, fruit juices containing flavonoids can inhibit OATP2B1 and reduce drug absorption (a notable example being grapefruit juice reducing fexofenadine uptake) [55,56].

PepT1 (SLC15A1) is another crucial uptake transporter, responsible for absorbing di- and tripeptides from protein digestion [57].

PepT1 is highly expressed in the small intestine (especially duodenum and jejunum) and operates as a proton-coupled symporter, using the H^+^ gradient to drive peptide uptake. Other SLC transporters in the gut include Organic Cation Transporters (OCTs, e.g., OCT3) and Organic Anion Transporters (OATs), though their roles in oral drug absorption are less prominent than OATPs and PepT1 [58,59].

Organic Cation Transporter OCT1 (SLC22A1) is present in the intestine and may contribute to the uptake of metformin and other cationic drugs, whereas OCTN2 (SLC22A5) aids absorption of carnitine and some drugs like mildronate. Monocarboxylate transporters (MCT1, SLC16A1) facilitate absorption of short-chain fatty acids and some drug analogs (like γ-hydroxybutyrate) by proton-linked transport. Each of these carriers has distinct substrate profiles and regional expression patterns, collectively ensuring efficient uptake of nutrients while also affecting drug entry [60].

Regional Expression and Coordinated Function: In the GI tract, transporter expression is not uniform. P-gp expression tends to increase from the proximal to distal small intestine (duodenum < ileum), whereas BCRP shows the highest expression in the mid to distal small intestine and colon [61]. PepT1 is abundant in the duodenum and jejunum (site of maximal peptide absorption) and declines towards the ileum and colon. These distributions align with the physiological roles of the transporters—for instance, PepT1 in the upper gut for capturing dietary peptides early, and P-gp/BCRP in the distal gut for limiting absorption of any residual or harmful compounds [62,63].

Known Age-Related Changes: Whether and how these transporter levels or activities change with age is a critical question. Historically, data have been somewhat conflicting, but recent analyses provide clearer insights. For P-glycoprotein, several studies indicate little change in intestinal P-gp expression between healthy younger and older adults. P-gp function “appears stable in healthy older adults”, corroborating earlier human biopsy studies that found no significant difference in intestinal P-gp mRNA/protein with age [64,65].

However, context matters: in older patients with comorbidities (e.g., chronic kidney disease), P-gp expression/function may be reduced, and certain genetic polymorphisms prevalent in the elderly can also affect P-gp levels [66]. In contrast to P-gp, BCRP (ABCG2) does seem to decline with age. Both intestinal BCRP expression and hepatic BCRP expression were reported to be lower in older individuals compared to young adults. This reduction could increase absorption of BCRP substrates and decrease their excretion [67,68].

Data on MRPs in aging are sparse; some rodent studies hint at decreased Mrp2 in the aged intestine, but human data are lacking. On the uptake side, information is limited. OATP2B1 and PepT1 have not been conclusively studied in the aged human intestine [69].

It is hypothesized that if intestinal surface area and enterocyte turnover decrease with age, there may be a proportional decrease in total transporter capacity for uptake, but specific gene expression changes remain unclear [70]. One intriguing observation from animal models is that PEPT1 might actually be preserved or even upregulated with age, possibly as a compensatory mechanism to maintain nutrition [71]. Table 1 summarizes these patterns, noting where evidence is strong (e.g., BCRP decrease) versus where data are insufficient (many SLCs).

**Table 1:** Key drug transporters in the gastrointestinal tract and known age-related changes.

Transporter (Gene)	Family (Type)	Major Substrates (Examples)	Localization in Gut	Reported Age-Related Changes	References
P-glycoprotein (*ABCB1/MDR1*)	ABC—Efflux pump	Broad spectrum of xenobiotics: e.g., digoxin, paclitaxel, calcineurin inhibitors, HIV protease inhibitors	Apical membrane of enterocytes (high in ileum)	Expression/function largely unchanged in healthy aging; may decrease with comorbidities (e.g., CKD).	[42,43,44,45,46,47,48,49]
BCRP (*ABCG2*)	ABC—Efflux pump	Anticancer drugs (e.g., irinotecan), statins (rosuvastatin), dietary toxins (heterocyclic amines)	Apical membrane of enterocytes (duodenum through colon)	Expression decreases with age in intestine and liver, potentially increasing substrate drug absorption.	[47,48,49,50]
MRP2 (*ABCC2*)	ABC—Efflux pump	Phase II conjugates (glucuronide, GSH conjugates), some antivirals (tenofovir), methotrexate	Apical membrane of enterocytes (upper small intestine)	Limited data; animal studies suggest possible decline in activity with age, but human age effect not well established.	[47,48,49]
MRP3 (*ABCC3*)	ABC—Efflux (basolateral)	Similar to MRP2 (conjugated bilirubin, drug glucuronides)	Basolateral membrane of enterocytes (ileum, colon)	Limited data in aging; no clear evidence of change (maintains efflux of conjugates into circulation).	[51,52]
OATP2B1 (*SLCO2B1*)	SLC—Uptake transporter	Organic anions: e.g., fexofenadine, atorvastatin, rosuvastatin, flavonoids	Apical membrane of enterocytes (duodenum > jejunum)	No direct human data on aging; expression assumed to be stable in adulthood. Potentially reduced function if gut surface area declines; needs investigation.	[55,56]
PepT1 (*SLC15A1*)	SLC—Uptake transporter	Dipeptides, tripeptides; peptide-like drugs (β-lactam antibiotics, ACE inhibitors)	Apical membrane of enterocytes (duodenum, jejunum; low in ileum/colon)	Unclear in aging; some evidence of maintained or slightly increased expression in older animals. Likely preserved to ensure nutrient (peptide) absorption.	[57,58,59]
OCT3 (*SLC22A3*)	SLC—Uptake transporter	Organic cations: e.g., metformin, neurotransmitters (histamine)	Apical and basolateral membranes (scattered along intestine)	Not well characterized; no specific aging data. Indirectly, age-related systemic changes (e.g., reduced perfusion) might affect cation absorption more than expression.	[59]
OCTN2 *(SLC22A5*)	SLC—Uptake transporter	Carnitine and some cationic drugs (e.g., mildronate)	Apical membrane (ileum predominantly)	No data on age effect; presumably unchanged in absence of disease.	[60]
MCT1 (*SLC16A1*)	SLC—Uptake transporter	Monocarboxylates: short-chain fatty acids, salicylate, γ-hydroxybutyrate	Apical membrane (widely in intestine)	No specific aging studies; function might be influenced by age-related microbiome changes (altering SCFA availability) rather than expression.	[60]

## 4. Molecular Mechanisms Linking Mitochondria to Transporter Regulation

Age-related mitochondrial dysfunction can lead to altered regulation of drug transporters in the GI tract. Mitochondria influence cellular function not only by providing ATP, but also by generating signaling molecules (like ROS, NAD^+^/NADH, Ca^2+^) and interacting with various cellular stress pathways. These signals can modulate the activity of transcription factors and kinases that ultimately govern the expression or function of transporter proteins [72,73].

In this section, we discuss several major pathways and factors at the nexus of mitochondrial status and transporter regulation (summarized in Table 2). We focus on energy-sensing pathways (e.g., AMPK/mTOR – mechanistic target of rapamycyn), redox-sensitive transcription factors (e.g., Nrf2, NF-κB, p53), and longevity regulators (e.g., sirtuins/FOXO) that have been implicated in controlling transporter genes. We also consider the direct impact of ATP availability on transporter activity. Cellular Energy Sensors (AMPK and mTOR): Mitochondria are the powerhouses of the cell; when they falter, cellular ATP levels may drop or AMP levels rise, activating AMP-activated protein kinase (AMPK).

AMPK is an energy sensor that shifts cells into energy-conservation mode when ATP is scarce [74]. Activation of AMPK has multiple downstream effects that could influence transporters. One relevant effect is on the mTOR pathway: AMPK activation inhibits mTOR (mechanistic target of rapamycin), which is a kinase that promotes protein synthesis and growth. Reduced mTOR activity might downregulate the translation of certain proteins, potentially including transporters, or it may induce autophagy that can degrade membrane proteins [75]. Moreover, AMPK has been shown to interact with nuclear receptors that control transporter expression. For example, there is evidence that AMPK activation can suppress the Pregnane X Receptor (PXR), a xenobiotic-sensing nuclear receptor that induces both drug-metabolizing enzymes (like CYP3A4) and transporters (like P-gp). If mitochondrial dysfunction chronically activates AMPK in enterocytes, it could therefore lead to reduced PXR-mediated transcription of transporters, tending to lower basal expression of P-gp or MRP2 [76,77,78,79,80].

Sirtuins and FOXO Factors: Sirtuins, particularly SIRT1, are NAD^+^-dependent deacetylases that link cellular metabolic state to transcriptional programs. SIRT1 activity is influenced by the NAD^+^/NADH ratio, which in turn is affected by mitochondrial metabolic function. In aging, NAD^+^ levels generally decline, potentially diminishing SIRT1 activity. SIRT1 is known to deacetylate and activate FOXO transcription factors (FOXO1, FOXO3a), which are crucial mediators of stress resistance and longevity [81]. FOXO1/3a can enhance the transcription of the ABCB1 gene encoding P-glycoprotein. The promoter of MDR1/ABCB1 contains binding sequences for FOXO factors; when FOXO1/3a are activated (deacetylated by SIRT1 under stress conditions), they bind to the MDR1 promoter and increase P-gp expression [82]. This mechanism is thought to operate in cancer cells under stress, but it likely exists in normal cells as well. SIRT1-FOXO signaling (often associated with good mitochondrial function and metabolic health) could upregulate P-gp, whereas in aging, a decline in SIRT1 or FOXO activity might remove this positive drive, potentially lowering P-gp expression [83]. Consistent with this, SIRT1 is typically lower in aged tissues, correlating with reduced FOXO target gene expression. Conversely, interventions that boost SIRT1 (like resveratrol or NAD^+^ precursors) could augment P-gp expression via FOXO—a possibility that is being explored in contexts like enhancing drug efflux to protect tissues from toxins. Excessive FOXO activation can have cell-type-specific effects; in some cells, it might tilt them toward pro-apoptotic genes. In enterocytes, moderate FOXO activity is generally considered protective and may maintain transporter levels as part of a detoxification program [84,85,86].

A dysfunctional mitochondrion is a major source of ROS, and aging cells often experience chronic oxidative stress. One critical responder to oxidative stress is the transcription factor nuclear factor erythroid 2-related factor 2 (Nrf2) [87]. Under stress, Nrf2 dissociates from its inhibitor Keap1 and translocates to the nucleus to bind antioxidant response elements (AREs) in target gene promoters. Nrf2 is classically known to induce Phase II detoxification enzymes (e.g., glutathione S-transferases, NAD(P)H quinone dehydrogenase 1) and antioxidant proteins (e.g., HO-1 – Heme Oxygenase-1, NQO1 – NAD(P)H Quinone Dehydrogenase 1). Importantly, Nrf2 also regulates the expression of several drug transporters. Studies have shown that activation of Nrf2 can upregulate MDR1 (P-gp), MRPs, and BCRP in various models [88,89].

For instance, genetic activation of Nrf2 in human cells led to higher expression of MDR1, MRP2/3, and BCRP, enhancing drug efflux capacity [90]. Mechanistically, Nrf2 binding sites have been identified in the promoters of transporter genes such as ABCG2 (BCRP) and ABCC2 (MRP2), directly linking oxidative stress response to transporter gene transcription [91]. In the intestine, a recent study demonstrated that abdominal X-ray irradiation (which induces ROS) caused Nrf2 activation and a significant increase in intestinal MRP2 expression, leading to reduced absorption of the MRP2 substrate valsartan [92]. NF-κB can act upstream to enhance Nrf2 in this context. Taken together, mitochondrial ROS production in aging could chronically activate Nrf2, which in turn could tend to elevate efflux transporter expression as a protective mechanism [93].

P-gp is unchanged or even reduced with age; however, it may be a matter of timing and degree [94]. In early aging or mild stress, Nrf2 upregulation might compensate by inducing transporters, whereas in advanced age, the Nrf2 pathway itself becomes less responsive (indeed, Nrf2 activity has been noted to decline in very old organisms despite high oxidative stress) [95]. Thus, an aging cell may initially attempt to upregulate transporters via Nrf2, but with chronic stress and eventual failure of adaptive responses, transporter expression could still decline. Another layer is that Nrf2 activity is modulated by diet and exercise—for example, certain phytochemicals (sulforaphane from broccoli) strongly activate Nrf2 and have been shown to increase P-gp and BCRP expression. In elderly individuals with poor diets, lower Nrf2-inducing compounds might further diminish this pathway. In summary, Nrf2 connects mitochondrial oxidative stress to transporter regulation by typically upregulating efflux transporters, although the net effect in aging depends on the integrity of Nrf2 signaling, which might be compromised with advanced age. Inflammation and NF-κB: Aging is often accompanied by a state of chronic low-grade inflammation (so-called “inflammaging”) [96,97,98].

Mitochondrial dysfunction can contribute to this by releasing damage-associated molecular patterns (e.g., mtDNA) that activate immune responses, or by ROS activation of pro-inflammatory pathways [99]. A central mediator of inflammatory gene expression is NF-κB (nuclear factor kappa-light-chain-enhancer of activated B cells). NF-κB is typically kept inactive in the cytoplasm by IκB; upon stimulation by inflammatory cytokines or ROS, it enters the nucleus and induces a host of genes (cytokines, adhesion molecules, etc.) [100]. NF-κB also plays a direct role in transporter regulation: it binds to the MDR1 promoter and enhances P-glycoprotein expression. In some cancer cell lines, constitutive NF-κB activity correlates with high P-gp levels and multidrug resistance. The mechanistic basis is that the MDR1 promoter contains NF-κB response elements, so when NF-κB is activated (as in inflammation), it can drive MDR1 transcription [101]. Consistently, experiments have shown that stimuli like tumor necrosis factor (TNF-α) or lipopolysaccharide (which activate NF-κB) lead to increased P-gp expression in intestinal cells. It may seem counterintuitive that pro-inflammatory signaling upregulates a detox transporter—one interpretation is that during inflammation, the body attempts to pump out xenobiotics and possibly host-derived toxic metabolites to protect tissues [102]. In aging, with elevated levels of inflammatory cytokines (e.g., IL-6, TNF-α often mildly elevated in older adults), there could be a persistent NF-κB activation in enterocytes. This suggests that inflammaging might actually induce certain efflux transporters like P-gp [103].

On the other hand, inflammation is known to downregulate some uptake transporters and metabolic enzymes (a phenomenon seen in acute inflammation or infection, where the body prioritizes defense over absorption) [104]. For example, inflammatory cytokines have been shown to suppress Oatp and Cyp3a expression in the liver and have possibly analogous effects in the gut [105]. So, long-lasting inflammation caused by mitochondrial stress can shift the balance in the intestine toward more drug efflux and less uptake, which reduces overall drug absorption. Interestingly, NF-κB and p53 (a tumor-suppressor protein) affect P-gp in opposite ways: NF-κB increases P-gp expression, while p53 can reduce it [106].

p53 is activated by severe DNA damage or stress (including oxidative stress); active wild-type p53 represses MDR1 transcription as part of a program to sensitize cells to apoptosis. In aged cells with substantial damage, if p53 is triggered, it might reduce transporter expression, steering the cell toward apoptosis rather than survival. However, in many aged tissues, p53 responses are blunted or p53 may accumulate in mutant forms. Notably, mutant or deficient p53 is associated with increased P-gp expression (as seen in certain tumors and in p53-null mice, which had elevated P-gp in tissues) [107].

Some aging theories propose that p53 function declines with age or that cells with p53 mutations are preferentially retained. This can eliminate p53-mediated repression of MDR1, leading to higher P-gp levels. As a result, moderate NF-κB-driven inflammation may increase P-gp expression, whereas severe stress can reduce it through p53 activation, unless p53 is dysfunctional. During aging, these opposing signals likely vary between cells over time [108].

Beyond gene regulation, mitochondria can directly influence transporter activity through ATP availability. ABC transporters such as P-gp, BCRP, and MRPs require ATP to function, so impaired mitochondrial energy production can limit efflux, especially under high transport demand [109]. Studies have shown that ABC transporters preferentially use mitochondria-derived ATP, and that boosting mitochondrial ATP production increases drug efflux, while inhibiting mitochondrial respiration reduces transporter activity [110]. Although these findings come mainly from cancer models, the principle likely applies more broadly. In aging enterocytes with dysfunctional mitochondria, local ATP levels near the apical membrane may be insufficient during active transport, making efflux pumps less efficient even if their expression is unchanged [111]. Similar age-related reductions in P-gp function have been observed in other tissues, such as the blood–brain barrier [112]. Moreover, mitochondrial dysfunction alters the AMP/ATP ratio and activates AMPK, which can acutely reduce P-gp activity by removing it from the plasma membrane. Thus, low cellular energy not only limits ATP supply for transporters but also actively signals the cell to downregulate energy-intensive processes like active drug efflux [113].

Mitochondria also help regulate calcium levels in cells, and calcium-dependent signaling can affect transporter expression, for example, by modifying NF-κB activity. In addition, aging and mitochondrial stress can change microRNA patterns; some of these microRNAs can reduce the production of transporters like P-gp. Even though direct evidence in aging is limited, these pathways likely play a role in age-related differences in transporter expression [114]. Table 2 provides a concise summary of the information discussed above, highlighting how age-related changes in mitochondrial function can influence the expression and activity of drug transporters.

**Table 2:** Mitochondrial pathways and signals influencing transporter regulation.

Mitochondrial/ Cellular Pathway	Effect on Transporters	Mechanism or Key Players	References
Energy status—AMPK/mTOR	Low ATP/high AMP (activating AMPK) tends to downregulate some transporters (particularly uptake) and can reduce transporter activity; high energy (mTOR active) supports protein synthesis (including transporters)	AMPK activation suppresses PXR-driven MDR1 expression; CR (which activates AMPK) increases P-gp expression in mice (perhaps via hormesis). AMPK may cause internalization of P-gp to conserve energy (seen in stress conditions).	[74,75,76,77,78,79,80]
Sirtuin–FOXO signaling	Generally upregulates efflux transporters (e.g., P-gp) under stress conditions that activate SIRT1/FOXO	SIRT1 (NAD^+^-dependent deacetylase) deacetylates and activates FOXO1/3a, which bind MDR1 promoter and enhance P-gp transcription. In high stress or caloric restriction, FOXO-driven detox programs (including transporters) are increased. With aging, lower NAD^+^ may reduce SIRT1/FOXO activity, potentially lowering transporter expression.	[81,82,83,84,85,86]
Oxidative stress—Nrf2 pathway	Induces many drug transporters (efflux pumps) as part of antioxidant response, though chronic activation may eventually exhaust pathway	Nrf2 activation (e.g., via ROS or electrophiles) upregulates ABCB1, ABCC2, and ABCG2, among others. Nrf2 binds AREs in transporter gene promoters (e.g., BCRP) and increases their transcription. Example: Oxidative stress (radiation) raised intestinal MRP2 and lowered drug absorption via Nrf2. In extreme age, Nrf2 responsiveness declines, possibly reducing this effect.	[87,88,89,90,91,92,93,94,95,96,97,98]
Inflammation—NF-κB and cytokines	NF-κB activation boosts P-gp (and possibly MRP) expression; pro-inflammatory cytokines often decrease uptake transporters and metabolic enzymes	NF-κB p65 subunit binds MDR1 promoter, increasing P-gp expression. Chronic IL-6/TNF in aging (“inflammaging”) may sustain higher baseline P-gp. However, cytokines like IL-1β and IL-6 can suppress OATP and PepT1 expression (observed in inflammatory states) to reduce absorption. Net effect: shift toward efflux over uptake. NF-κB also crosstalks with Nrf2 (can augment Nrf2).	[100,101,102,103]
p53 and stress/apoptosis	Active wild-type p53 represses certain transporter genes; loss of p53 (or mutant p53) removes this repression, allowing high transporter expression	p53 binds to MDR1 promoter and downregulates P-gp expression as part of pro-apoptotic program. In aging cells with significant DNA damage, transient p53 activation might reduce transporter levels (favoring drug retention and cell death). However, aged cells often accumulate p53 mutations or have dysfunctional p53, which has been linked to elevated P-gp (seen in tumors and p53-null models). Thus, cells that escape apoptosis (e.g., via p53 loss) may overexpress transporters.	[107,108]
Mitochondrial ATP supply	High mitochondrial ATP supports maximal efflux pump activity; ATP depletion impairs transporter function	ABC transporters require ATP—studies show that they preferentially use mitochondrial-produced ATP at the membrane. Enhanced mitochondrial respiration (e.g., via loss of MCJ protein) increased drug efflux capacity. In aging, reduced ATP can directly limit P-gp/BCRP pumping ability. Additionally, low ATP/high AMP can trigger AMPK to reduce energy-consuming processes (transporter cycling).	[109,110,111,112,113]
Mitochondrial metabolites (NAD^+^, acetyl-CoA)	Alter gene regulation epigenetically, impacting transporters indirectly	NAD^+^ levels modulate sirtuins (as above). Acetyl-CoA levels affect histone acetylation: energy surplus increases global acetylation (could enhance expression of some genes), while energy deficit reduces it (silencing some genes). Transporter genes could be among those affected by age-related epigenetic changes due to metabolite shifts. For example, age-related hypermethylation or altered acetylation at transporter promoters might reduce expression (noted in some ABC transporters in cancer/aging).	[81]
Microbiome–metabolite signaling	Gut microbial metabolites (e.g., bile acids, short-chain fatty acids) modulate nuclear receptors that regulate transporters	Mitochondrial dysfunction can alter gut microbiota composition. In turn, microbiota-derived molecules activate receptors like FXR, PXR, and AHR. Bile acids (changed in aging) via FXR can repress or induce certain transporters (FXR induces intestinal BSEP, affects P-gp indirectly). SCFAs via HIF-2α signaling might enhance PepT1 for increased nutrient uptake. Aging microbiota changes could thus lead to secondary changes in transporter regulation. (This area is still emerging, with no consensus data yet.)	[115]

## 5. Pharmacokinetic Implications in the Elderly

Age-related changes in drug transporters and the underlying mitochondrial-regulatory mechanisms are not merely academic—they can translate into observable differences in drug disposition and response in older patients [116]. The pharmacokinetic (PK) implications of these changes manifest most clearly in the absorption phase for orally administered drugs. If an older individual has altered expression or function of intestinal transporters, the fraction of an oral dose that reaches systemic circulation (bioavailability) may increase or decrease relative to a younger individual [117].

This can lead to a higher risk of toxicity or, conversely, sub-therapeutic levels, depending on the direction of change. Additionally, changes in efflux transporters at barrier sites such as the intestine or blood–brain barrier can affect drug distribution and elimination [115].

## 6. Oral Drug Absorption—General Trends

Despite age-related physiological changes in the GI tract, it has long been observed that intestinal drug absorption is often relatively preserved in healthy older adults, especially for drugs absorbed by passive diffusion. However, drugs that rely on transporters or undergo extensive intestinal first-pass effects do show variability. Aging can cause drug-specific changes—some drugs have higher exposure in the elderly while others are unchanged [118]. This is consistent with the idea that transporter function may be altered for certain substrates (e.g., if an efflux mechanism is impaired, more drug is absorbed, raising exposure). For drugs with high first-pass extraction in the gut (meaning that normally, a large portion is pumped out or metabolized in the intestinal wall), an age-related reduction in these processes will increase bioavailability. By contrast, if an uptake transporter is downregulated, absorption might decrease for its substrates [119].

One well-documented example is fexofenadine, a non-sedating antihistamine that is a substrate of both OATP uptake and P-gp efflux in the gut [120]. Clinical studies have found that in geriatric patients (≥65 years), fexofenadine plasma levels are significantly higher than in younger adults—peak concentrations are about 99% higher on average. In other words, C_max was nearly double in the older group, and AUC was also elevated [121]. This suggests increased bioavailability of fexofenadine in the elderly, likely due to diminished efflux and/or enhanced uptake. Given that BCRP and possibly P-gp are downregulated with age, the intestinal efflux of fexofenadine may be less effective, allowing more absorption [122]. Additionally, if there is any reduction in OATP2B1 with age (unconfirmed, but possible), one might expect lower absorption; the fact that absorption is higher implies efflux decline is the dominant factor here. Clinically, this does not usually necessitate dose adjustment for fexofenadine, as the drug has a wide safety margin [123]. However, it serves as a probe for P-gp function in studies—indeed, fexofenadine has been used as a probe substrate to gauge intestinal P-gp activity, and elevated fexofenadine levels in older patients are taken as evidence of reduced P-gp function with age [124].

Another classic example is digoxin, a cardiac glycoside and narrow-therapeutic-index drug, which is a known P-gp substrate. The pharmacokinetics of digoxin are markedly influenced by P-gp: intestinal P-gp limits its absorption, and renal P-gp mediates its excretion. In older individuals, especially those over 75 or with frailty, studies have reported higher digoxin plasma concentrations and a prolonged half-life [125]. Part of this is due to decreased renal function with age (digoxin is renally cleared). However, even after accounting for glomerular filtration rate, reduced P-gp-mediated renal and biliary secretion in aging may further elevate digoxin levels [126]. Aging is associated with tissue-specific changes: intestinal P-gp may not increase with age, and renal P-gp actually decreases, so overall elimination is slower [127]. Additionally, if intestinal P-gp expression or activity is slightly diminished in the elderly, digoxin’s fraction absorbed could rise. The net effect is a higher risk of digoxin toxicity in older patients if doses are not adjusted [128]. Indeed, standard geriatric practice is to use lower digoxin doses and monitor levels carefully, reflecting this pharmacokinetic reality [129].

Calcium channel blockers like verapamil: Verapamil is metabolized by CYP3A and also a P-gp substrate. Older adults often have reduced intestinal CYP3A activity (some reports suggest ~50% lower CYP3A expression by the eighth decade) [130]. If P-gp is also reduced or unchanged, the overall first-pass extraction is less, meaning that more verapamil reaches circulation, which can cause exaggerated blood pressure lowering or bradycardia in an older patient [131]. However, verapamil’s case is complicated by its hepatic metabolism changes too. The general principle stands: any reduction in the “barriers” to absorption (metabolic enzymes or efflux pumps) will increase systemic drug exposure [132].

On the other hand, some drugs may have decreased absorption in the elderly due to transporter-related issues. A possible case is medications requiring active uptake that is compromised. For example, certain vitamin and mineral uptakes decline—vitamin B12 malabsorption is common, although it involves intrinsic factors more than transporters [133,134]. One drug parallel is levodopa, absorbed via amino acid transporters in the gut; competition with dietary amino acids and slowed gastric emptying in older patients can reduce its absorption [135]. While not an ABC/SLC transporter alteration per se, this highlights that age changes in GI physiology can modulate apparent transporter function (slower transit might allow more time for efflux) [136].

Acyclovir is absorbed via PepT1 when given as a prodrug (valacyclovir, famciclovir), and requires adequate PepT1 function. If an older adult has protein malnutrition or mucosal atrophy, PepT1 activity might be lower, potentially reducing prodrug absorption. There is limited clinical data on this, possibly because dosing compensates or these changes are minor [137,138,139].

One interesting and clinically important category is direct oral anticoagulants (DOACs), such as dabigatran etexilate and rivaroxaban, which are P-gp substrates. Dabigatran etexilate (a prodrug of dabigatran) is absorbed via P-gp; in fact, P-gp is so crucial that co-administration of a P-gp inhibitor (like dronedarone or clarithromycin) can double dabigatran levels and bleeding risk [140,141]. The elderly are at baseline increased risk of bleeding, and indeed, guidelines often recommend dose reduction in dabigatran in patients ≥75 or those with impaired renal function [142]. If we consider an older patient with somewhat reduced P-gp in the gut, dabigatran absorption could be increased, compounding bleeding risk [143]. Although formal studies isolating age vs. P-gp effects are few, it is telling that dabigatran exposure varies widely and that the highest levels are often seen in older patients [144]. The advice to avoid P-gp inhibitors in elderly patients on dabigatran underscores how critical transporter function is in that context [145]. Similarly, rivaroxaban and apixaban (factor Xa inhibitors) are substrates of P-gp and BCRP; their PK might be altered by age-related changes in these transporters as well as organ clearance [146]. In fact, apixaban’s bioavailability is high (~50%), but any further increase in the elderly (due to less efflux) plus decreased clearance can significantly raise plasma levels, so careful dosing is warranted in older patients, especially those with polypharmacy affecting P-gp [147].

Statins such as atorvastatin, simvastatin, and rosuvastatin rely on transporters for both hepatic uptake (OATP1B in liver) and intestinal absorption (OATP2B1, P-gp, BCRP). Aging is known to reduce hepatic OATP expression in some studies, which can increase statin plasma levels (since less is taken up by liver for clearance) [148,149]. Intestinally, the drop in BCRP with age means that statins that are BCRP substrates (notably rosuvastatin and atorvastatin) may have higher bioavailability in older patients. Indeed, rosuvastatin exposure has been observed to be somewhat higher in the elderly, and interestingly, polymorphisms in ABCG2 that reduce BCRP function mimic this by raising rosuvastatin levels and risk of muscle toxicity. Myopathy risk with statins is reported to be higher in older adults, which is likely multifactorial (including reduced muscle mass, co-medications, etc.), but higher plasma concentrations due to transporter and metabolic changes contribute. This is one reason that clinicians start at lower statin doses in elderly patients and monitor for adverse effects [150,151,152].

Beyond absorption, transporter changes can affect distribution and elimination. For instance, P-gp at the blood–brain barrier (BBB) tends to decline in function with age. This is not directly related to GI absorption, but it means that older adults might have more drug crossing into the CNS [153,154]. A practical example is loperamide, an opioid antidiarrheal that is normally kept out of the brain by P-gp. Elderly patients, especially if on a P-gp inhibitor or if P-gp is naturally low, could experience central opioid effects such as sedation or even respiratory depression from loperamide, something virtually never seen in younger individuals [155]. Another example is metoclopramide, a prokinetic that is a P-gp substrate; increased BBB penetration in older people, together with reduced renal clearance, is thought to contribute to a higher incidence of extrapyramidal side effects in the elderly [156]. The comprehensive summary can be found below (Table 3).

Inter-Individual Variability: It must be stressed that aging is not a uniform process. The studies we cite often report population averages—e.g., “on average, double fexofenadine levels in ≥65 vs. <65”—but within these older groups, some individuals had levels similar to young ones while others had much higher levels. This variability likely reflects differences in the biological age of the gut wall, genetic differences in transporter expression (polymorphisms in ABCB1, ABCG2, etc.), and environmental factors (diet, microbiome, disease) [157,158].

Some older adults maintain a relatively healthy gut with intact mitochondrial function (perhaps due to lifelong exercise or caloric moderation), thus retaining robust transporter activity, whereas others with multiple comorbidities (heart failure, diabetes) might have a more “aged” gut with reduced transporter function [159]. This variability can make it difficult to generalize PK changes—it explains why some studies find no significant age effect for a given drug, whereas others do when looking at a frailer cohort [160].

It also underlines the importance of therapeutic drug monitoring (TDM) for drugs with narrow therapeutic indices in older patients. For example, measuring digoxin levels, or antiarrhythmic levels, can catch individual differences that an age-based dosing guideline might miss [161].

**Table 3:** This table provides concrete drug examples with their transporters and observed changes to reinforce the points made above.

Drug (Class)	Key Transporter(s) Involved	Age-Related PK Change	Notes/Mechanism	References
Digoxin (cardiac glycoside)	P-gp (ABCB1) efflux in gut and kidney	Higher plasma levels and longer half-life in older adults. Elderly often require dose reduction.	Lower P-gp activity in intestines (↑ absorption) and kidneys (↓ excretion) contributes to increased exposure. Age-related renal decline also a factor. Monitor levels in elderly.	[125,126,127,128,129]
Fexofenadine (antihistamine)	P-gp (efflux); OATP2B1 (uptake)	~Two-fold higher C_max and AUC in ≥65 vs. young patients.	Likely due to reduced intestinal efflux (P-gp/BCRP) in older adults, allowing more drug to absorb. No major toxicity issue, but illustrates diminished efflux capacity.	[120,121,122,123,124]
Rosuvastatin (statin)	BCRP (ABCG2) efflux; OATP uptake (hepatic and intestinal)	Tend toward higher statin plasma concentrations in older patients (↑ risk of myopathy).	Decreased BCRP in gut and liver with age reduces rosuvastatin clearance (both absorption phase and hepatic elimination). Start low doses in elderly and titrate carefully.	[148,149,150,151,152]
Dabigatran etexilate (DOAC prodrug)	P-gp efflux (limits absorption)	Potentially increased bioavailability in older patients; significantly higher levels if combined with P-gp inhibitors.	Aging-related P-gp decline in gut may enhance dabigatran absorption. This, plus reduced renal function, raises bleeding risk. Dose adjustment and avoidance of strong P-gp inhibitors in ≥75 recommended.	[140,141,142,143,144]
Loperamide (opioid antidiarrheal)	P-gp (efflux in gut and BBB)	Greater CNS effects in some older patients (e.g., sedation) compared to virtually none in young patients.	Normally, P-gp keeps loperamide out of brain. If P-gp at BBB is reduced in aging, loperamide can cross and cause central opioid effects. Also, if intestinal P-gp is lower, systemic absorption of loperamide increases. Caution in elderly, especially if on other P-gp inhibitors.	[155]
Metformin (biguanide antidiabetic)	OCT3, OCT1 (uptake in gut and liver); MATE1/2 (renal efflux)	Absorption may be slightly delayed/reduced (due to slower GI motility), but total AUC is not drastically changed; however, accumulation risk is associated with reduced renal function.	Not a clear transporter–aging example for absorption; included to note that metformin uses SLC transporters (OCTs) for distribution. Any age effect is overshadowed by renal clearance decline. Shows that not all transporter substrates have significant age PK changes—depends on which step is rate-limiting [162,163].	[125,126,127,128,129]
Morphine (opioid analgesic)	P-gp (minimal at BBB for morphine; mainly passive crossing)	Increased sensitivity in elderly due to PD changes; PK changes mainly due to reduced clearance.	Morphine listed to distinguish from loperamide: morphine crosses BBB largely by diffusion (not P-gp substrate to significant degree), so its increased effects in elderly are not transporter-related but rather pharmacodynamic and metabolic (lower metabolism) [164,165].	[153,154,155]

## 7. Therapeutic Considerations and Interventions

The convergence of aging, mitochondrial dysfunction, and altered transporter function has several practical implications for clinical therapy in older adults. Recognizing these links enables a more informed approach to prescribing, often termed “geriatric pharmacology” [166].

Given that aging can increase bioavailability of certain drugs, a straightforward step is dose reduction for drugs with a narrow therapeutic index that are known transporter substrates. For example, clinicians often start at a lower dose of digoxin, certain anticoagulants (like DOACs), or CNS-active drugs in older patients. This practice, borne out of experience, aligns with the mechanistic understanding that reduced P-gp or BCRP function might elevate drug levels [167].

Additionally, employing therapeutic drug monitoring (TDM) for drugs like digoxin, cyclosporine, or tacrolimus in older patients is prudent [168]. If an elderly patient on digoxin presents with symptoms of toxicity (nausea, confusion) despite a “normal” dose, measuring plasma digoxin can reveal whether they have higher levels than expected, possibly due to their transporters and clearance not handling the drug as in a young adult [169]. TDM can thus catch those outliers with more pronounced transporter decline. For drugs without easy TDM, close clinical monitoring for signs of toxicity or inadequate effect is important when initiating therapy or changing doses in the elderly [170]. Polypharmacy is common in geriatrics, raising the risk of pharmacokinetic DDIs, especially those involving transporter inhibition or induction [171]. Many medications, including antiarrhythmics, antifungals, antivirals, and even supplements, can inhibit or induce P-gp and other transporters. In an older patient, a transporter DDI can have a magnified effect [172]. For instance, co-prescribing verapamil (a P-gp inhibitor) with dabigatran in an 80-year-old could lead to a sharp increase in dabigatran levels and bleeding risk [173].

Leveraging Interventions to Improve Mitochondrial Function: A novel angle is whether improving mitochondrial health in the elderly could secondarily normalize transporter function and pharmacokinetics. This falls under the concept of “geroprotective” or “mitochondria-targeted” therapies. Several approaches are being explored:

1. Exercise: Regular aerobic exercise is one of the most effective ways to enhance mitochondrial function in various tissues, including skeletal muscle and potentially the gut. Animal studies showed that exercise can restore some deficits in aging colonic mitochondria [174]. Although direct evidence in humans for intestinal transporter changes with exercise is not available, exercise does improve overall circulation and metabolic fitness, which might support better GI function. Anecdotally, fitter older adults often tolerate medications better, possibly due to these physiological reserves. Encouraging exercise (as tolerated) in older patients might thus indirectly maintain transporter expression via improved a mitochondrial and metabolic milieu (increased AMPK/SIRT1 from exercise might keep P-gp upregulated, for instance) [175].
2. Caloric Restriction (CR) or Dietary Modulation: CR is known in model organisms to preserve mitochondrial function and induce hormetic stress responses, including upregulation of detoxification pathways. While prolonged CR is not practical or safe for many elderly individuals, who may be frail or at risk of malnutrition, a mild reduction in overnutrition and emphasis on a nutrient-dense diet could be beneficial [176,177,178].
  Moreover, specific dietary components can activate protective pathways. For example, polyphenols such as resveratrol, curcumin, and sulforaphane activate Nrf2 and sirtuins. Including such compounds through diet or supplements might strengthen antioxidant defenses and possibly support transporter expression. For instance, resveratrol has been shown to activate SIRT1 and may increase P-gp via FOXO, in addition to its other biological effects [179,180].
  At the same time, caution is required. Grapefruit juice, another polyphenol-rich dietary component, acutely inhibits OATPs and P-gp and can lead to clinically relevant drug–drug interactions. Therefore, if dietary strategies are used to support mitochondrial or transporter function, they should be applied consistently and with a clear understanding of their interaction potential [181,182].
3. Mitochondria-Targeted Therapies: These are an exciting frontier. Compounds like mitoquinone (MitoQ) and elamipretide (SS-31) are designed to concentrate in mitochondria and reduce oxidative stress or improve electron transport. Trials of such agents in conditions like heart failure or frailty are underway. If effective, one could speculate that they might also benefit the aging gut mucosa. By reducing mitochondrial ROS, they could tone down pathological Nrf2/NF-κB activation, potentially normalizing any aberrant transporter induction or suppression [183,184]. For example, lowering oxidative stress might prevent the inflammation-driven decrease in uptake transporters. Additionally, compounds boosting NAD^+^ levels (like nicotinamide riboside or NMN) aim to enhance sirtuin activity in aging; these could, in theory, maintain SIRT1-FOXO-mediated P-gp expression, as seen in younger individuals [185,186].
4. Pharmacological Modulators of Transporters: In some cases, co-administering a transporter inhibitor or inducer deliberately can be therapeutic. For instance, in multidrug-resistant HIV or cancer, P-gp inhibitors have been used to increase drug levels [187]. In an aging context, one might consider whether inducing a transporter could lower toxicity (e.g., inducing P-gp to reduce loperamide CNS entry in an older patient with chronic diarrhea—although no specific inducers are used clinically for this purpose) [188]. Another example is rifampicin, which induces P-gp and CYP enzymes. In an older patient, this can markedly lower the levels of many drugs. Depending on the situation, this may be harmful by causing underdosing, or occasionally helpful if the goal is to reduce toxicity. In general, deliberately changing transporter activity with additional drugs is risky in elderly patients because responses are often unpredictable. For this reason, such approaches are usually limited to special situations. In everyday practice, it is safer to adjust the dose or formulation of the main drug rather than try to modify transporter function with another medication [189].
5. Drug Formulation and Route of Administration: If GI absorption is deemed too unpredictable or significantly altered by aging, alternative routes or formulations can be considered. For example, drugs with poor or variable oral absorption in older patients might be given transdermally or intravenously. Morphine bioavailability can be erratic in the elderly (due to metabolism differences), so clinicians sometimes use transdermal fentanyl or buprenorphine patches for chronic pain—these bypass first-pass and transporter issues (though they have their own considerations) [190,191]. Levodopa intestinal gel (duodenal infusion) is used in advanced Parkinson’s, partly to overcome absorption fluctuations. While not directly related to transporters, this highlights tailoring drug delivery in the elderly [192].
6. Managing Inflammaging and GI Health: Since chronic low-grade inflammation can modulate transporters, interventions aimed at reducing systemic and gut inflammation might help stabilize transporter expression [193]. This overlaps with the diet/exercise points (as these reduce inflammation too), but could also include ensuring adequate vitamin D (some evidence suggests that vitamin D can upregulate P-gp at the blood–brain barrier and maintain tight junctions; deficiency is common in the elderly), probiotics or prebiotics to improve gut barrier function (a healthier microbiome might produce metabolites that favor normal transporter regulation, e.g., butyrate, which nourishes colonocytes and could indirectly support MCT1 and PepT1 function) [194,195]. Additionally, avoiding Non-steroidal Anti-Inflammatory Drugs (NSAIDs) or other mucosal irritants when possible helps maintain an intact mucosa—ulcerations or gastritis in older patients can alter drug absorption unpredictably [196].

## 8. Conclusions

The interaction between aging, mitochondrial dysfunction, and drug transporter activity in the gastrointestinal tract is an important and rapidly developing area of research with clear clinical relevance. As discussed above, aging is linked to a series of changes: Mitochondria in intestinal cells become less efficient and produce more oxidative stress. This then activates both protective and harmful cell responses, which can change how important drug transporters are produced and how well they work. Transporters from the ABC and SLC families act as gatekeepers of drug absorption, so age-related changes in their activity help explain why older adults often show altered drug pharmacokinetics and different responses to medications.

At the molecular level, we described how energy-sensing pathways (AMPK/mTOR), stress-responsive transcription factors (FOXO, Nrf2, NF-κB, p53), and related signaling networks connect mitochondrial function with transporter regulation. These mechanisms help unify earlier clinical observations, such as increased oral bioavailability of certain drugs in older people, by linking them to transporter changes driven by the physiology of aging cells. An important concept is that mild mitochondrial and oxidative stress during aging may initially increase efflux transporter expression as a protective response, whereas severe or long-lasting dysfunction can eventually cause transporter loss and reduced function. The final outcome in any individual older patient depends on the balance between these opposing processes and is further shaped by genetics and coexisting diseases.

From a clinical pharmacology perspective, understanding these processes helps clinicians manage drug therapy more effectively in older adults. Dose adjustments, closer monitoring for both efficacy and toxicity, and avoidance of high-risk drug combinations are practical steps that follow directly from recognizing the link between aging and transporter function. There is also growing interest in new approaches: preserving mitochondrial health through lifestyle measures or targeted therapies may indirectly stabilize transporter activity and reduce adverse drug reactions in the elderly. For example, strategies that activate Nrf2 or SIRT1 could help maintain transporter expression at levels closer to those seen in younger individuals, while anti-inflammatory and antioxidant approaches might prevent abnormal increases or decreases in transporter expression caused by chronic stress. Although these ideas still need to be confirmed in clinical trials, they offer new ways to improve drug handling in older patients beyond standard pharmacokinetic adjustments.

First, there is a clear need for biomarkers that could inform, at the level of an individual patient, how the intestinal barrier and drug transporters are functioning, without the need for invasive biopsies. Potential candidates include blood- and stool-based panels encompassing markers of oxidative stress and inflammation (such as lipid peroxidation products or cytokine signatures) and indicators of epithelial barrier disruption, as well as metabolomic profiles related to the NAD^+^/NADH redox state and microbiome-derived metabolites.

Second, from a translational perspective, the concept of “transporter phenotyping” in older adults using safe probe drugs administered at microdoses is particularly attractive. Such an approach would allow functional assessment of intestinal efflux and uptake capacity in vivo, rather than relying solely on gene or protein expression data.

Third, personalized medicine in this area is likely to benefit from integrating genetic information with functional phenotyping. While polymorphisms in ABCB1 and ABCG2 are known to influence drug exposure, genetics alone is often insufficient in older adults, where comorbidities, nutritional status, concomitant medications, and the gut microbiome substantially modify transporter function. A promising research direction is therefore the development of a “layered model” combining genotype (*ABCB1*/*ABCG2*/*SLCO*/*SLC15A1*), markers of mitochondrial function, probe-drug-based transporter phenotyping, and clinical variables such as frailty, chronic kidney disease, and heart failure. Such an integrated framework could bring us closer to predicting individual oral bioavailability and the risk of adverse drug reactions.

Fourth, an important translational question is whether improving mitochondrial function in the aging intestine translates into more predictable pharmacokinetics. Although conceptually straightforward, this hypothesis requires well-designed interventional studies with pharmacokinetic endpoints, in which changes in mitochondrial health are directly linked to alterations in drug absorption and exposure.

Fifth, aging is accompanied by shifts in gut microbiota composition and in the pool of microbial metabolites that can modulate transporter expression. This opens two complementary research avenues: observational studies linking microbiome profiles with transporter function, and interventional studies using prebiotics, probiotics, or fiber-rich diets in which transporter activity and drug bioavailability are included as outcome measures. Such approaches may help explain part of the inter-individual variability observed in older patients that is currently attributed to “random” differences.

Sixth, polypharmacy represents a major clinical challenge in geriatrics. A natural translational direction is the development of tools capable of predicting the combined impact of multiple drugs on P-gp, BCRP, and SLC transporters in the context of aging, taking into account not only classical drug–drug interactions but also age-related changes in cellular energy status and inflammatory tone.

If biomarkers, probe-drug strategies, and physiologically based pharmacokinetic (PBPK) modeling can be successfully integrated, a realistic framework for dose individualization in geriatric pharmacotherapy may emerge. In such a paradigm, chronological age would be replaced by a set of measurable biological characteristics—intestinal barrier integrity, bioenergetic status, and transporter phenotype—that can be directly leveraged to guide therapeutic decision-making.

Overall, the connection between aging, mitochondrial dysfunction, and gastrointestinal transporter function clearly shows how gerontology, cell biology, and pharmacology intersect. Understanding this relationship helps explain why older adults represent a unique group in pharmacotherapy and highlights the value of mechanism-based strategies for improving their clinical care. As the global population continues to age, ongoing research and awareness in this area will be essential to ensure safe and effective medication use. The long-term goal is truly rational drug therapy for older adults, therapy that fully accounts for the molecular and physiological changes that come with aging—a goal that the insights from this review help bring closer to reality.

## Data Availability

No new data were created or analyzed in this study. Data sharing is not applicable.
